# Ability of SPP1 to Alleviate Post‐Intracerebral Hemorrhage Ferroptosis via Nrf2/HO1 Pathway

**DOI:** 10.1002/brb3.70493

**Published:** 2025-05-05

**Authors:** Pengpeng Li, Zhenxin Tao, Yangyang Gao, Zhengqian Mu, Jiajia Tian, YaTing Zhang, Wenhui Yang, Yilu Li, Xudong Zhao

**Affiliations:** ^1^ Wuxi School of Medicine Jiangnan University Wuxi Jiangsu China; ^2^ Department of Neurosurgery Jiangnan University Medical Center Wuxi Jiangsu China; ^3^ Department of Neurosurgery Medical School of Nantong University Nantong University Nantong Jiangsu China; ^4^ Department of Neurosurgery Ningxia Medical University Yinchuan China; ^5^ Department of Neurosurgery The Affiliated Wuxi No. 2 People's Hospital of Nanjing Medical University Wuxi Jiangsu China; ^6^ Department of Neurosugery Clinical College of Nantong University Wuxi Jiangsu China; ^7^ Wuxi Neurosurgical Institute Wuxi School of Medicine Jiangnan University Wuxi Jiangsu China

**Keywords:** brain injury, ferroptosis, ICH, OPN, SPP1

## Abstract

**Purpose:**

This study aimed to investigate the role of secretory phosphoprotein 1 (SPP1/OPN) in modulating iron‐induced cell death (ferroptosis) following intracerebral hemorrhage (ICH). By integrating transcriptomic analysis and experimental validation, we sought to identify key molecular pathways and therapeutic targets associated with ferroptosis in ICH.

**Method:**

The Gene Expression Omnibus Series GSE24265 dataset was analyzed using the limma package (R platform) to identify differentially expressed genes. Kyoto Encyclopedia of Genes and Genomes (KEGG) pathway and Gene Ontology (GO) enrichment analyses were performed to elucidate biological functions. Genes associated with iron‐induced mortality were identified by cross‐referencing transcriptomic profiles with the FerrDb database. A protein–protein interaction network was constructed using Cytoscape, and hub genes were identified. An experimental ICH model was developed in mice using stereotactic instrumentation, and the effects of OPN administration were evaluated through neurological assessments, biochemical assays (superoxide dismutase, glutathione, malondialdehyde), Western immunoblotting (GPX4, ACSL4), Prussian blue histochemistry, and electron microscopy.

**Finding:**

Transcriptomic analysis identified 27 hub genes, with CD44 and ITGB3 characterized as receptors for OPN. In the ICH model, OPN administration improved neurological outcomes, elevated antioxidant markers, and reduced lipid peroxidation. OPN upregulated GPX4 while suppressing ACSL4, indicating anti‐ferroptotic effects. These protective effects were mediated through the Nrf2 pathway, as confirmed by inhibitor ML385. Prussian blue staining and electron microscopy demonstrated reduced cerebral iron deposition and mitochondrial damage following OPN treatment.

**Conclusion:**

This study provides novel evidence for SPP1/OPN as a key modulator of ferroptosis in ICH, highlighting its potential as a therapeutic target. By enhancing iron homeostasis and mitigating oxidative stress, OPN offers a promising strategy for improving outcomes in ICH patients.

## Graphical abstract

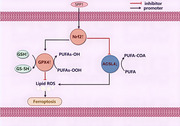

## 1 Introduction

More than 5 million cases of intracerebral hemorrhage (ICH) occur annually worldwide (Hanley et al. 2019). An estimated 10%–15% of strokes are caused by ICH, which carries high morbidity and mortality rates (Chen et al. 2022). Although surgery and blood pressure control have been utilized to treat ICH, no effective treatment is currently available (Zhao et al. 2017). Ferroptosis, an emerging form of regulated cell death that is distinct from apoptosis, is characterized by iron‐mediated peroxidation of cellular phospholipids. This process is characterized by the accumulation of lipid reactive oxygen species due to the dysregulation of iron metabolism and inhibition of antioxidant defenses, leading to cell death (Luo et al. 2022). Once red blood cells dissociate and disintegrate after ICH, a substantial amount of brain‐toxic iron is released into the brain parenchyma, resulting in neuronal death, disability, and other unfavorable outcomes (Karuppagounder et al. 2016). The excess iron produced during disintegration can induce the Fenton reaction, ultimately leading to ferroptosis (Sun et al. 2023). GPX4 is a recognized barrier to ferroptosis, whereas ACSL4, a lipid metabolism enzyme, promotes ferroptosis (Chen et al. 2021).

Secreted phosphoprotein 1 (SPP1), also known as osteopontin (OPN), is a multifunctional glycoprotein that plays crucial roles in human physiology and pathology (Karuppagounder et al. 2016). OPN engages with a variety of receptors and functions as a multifaceted adhesion molecule and cytokine and interacts with integrins and different isoforms of CD44, among other receptors, to modulate cellular responses (Zhou et al. 2020). OPN exhibits neuroprotective properties and shows promise as a therapeutic agent against acute brain injury (Zhou et al. 2020). Current evidence demonstrates that OPN facilitates tumor spheroid formation and angiogenesis in triple‐negative breast cancer (TNBC) through modulation of GPX4‐mediated anti‐lipid peroxidation via activation of the PI3K/AKT/mTOR signaling axis (Guo et al. 2024). Notably, while the regulatory role of OPN in ferroptosis has been established in TNBC models, its potential involvement in iron‐dependent cell death pathways following intracerebral hemorrhage remains unexplored in existing literature.

Here we employed R software in conjunction with the limma software package to analyze the GSE24265 dataset obtained from the Gene Expression Omnibus (GEO) database and identify differentially expressed genes (DEGs). We subsequently conducted enrichment analyses, with particular emphasis on the intersection between DEGs and the genes associated with ferroptosis, to elucidate the key pathways involved. By leveraging the Molecular Complex Assay (MCODE) plug‐in of Cytoscape software, we delineated key gene modules and identified key genes related to ferroptosis in ICH. Notably, the genes of interest were *CD44* and *ITGB3*, which serve as receptors for OPN.

**FIGURE 1:**
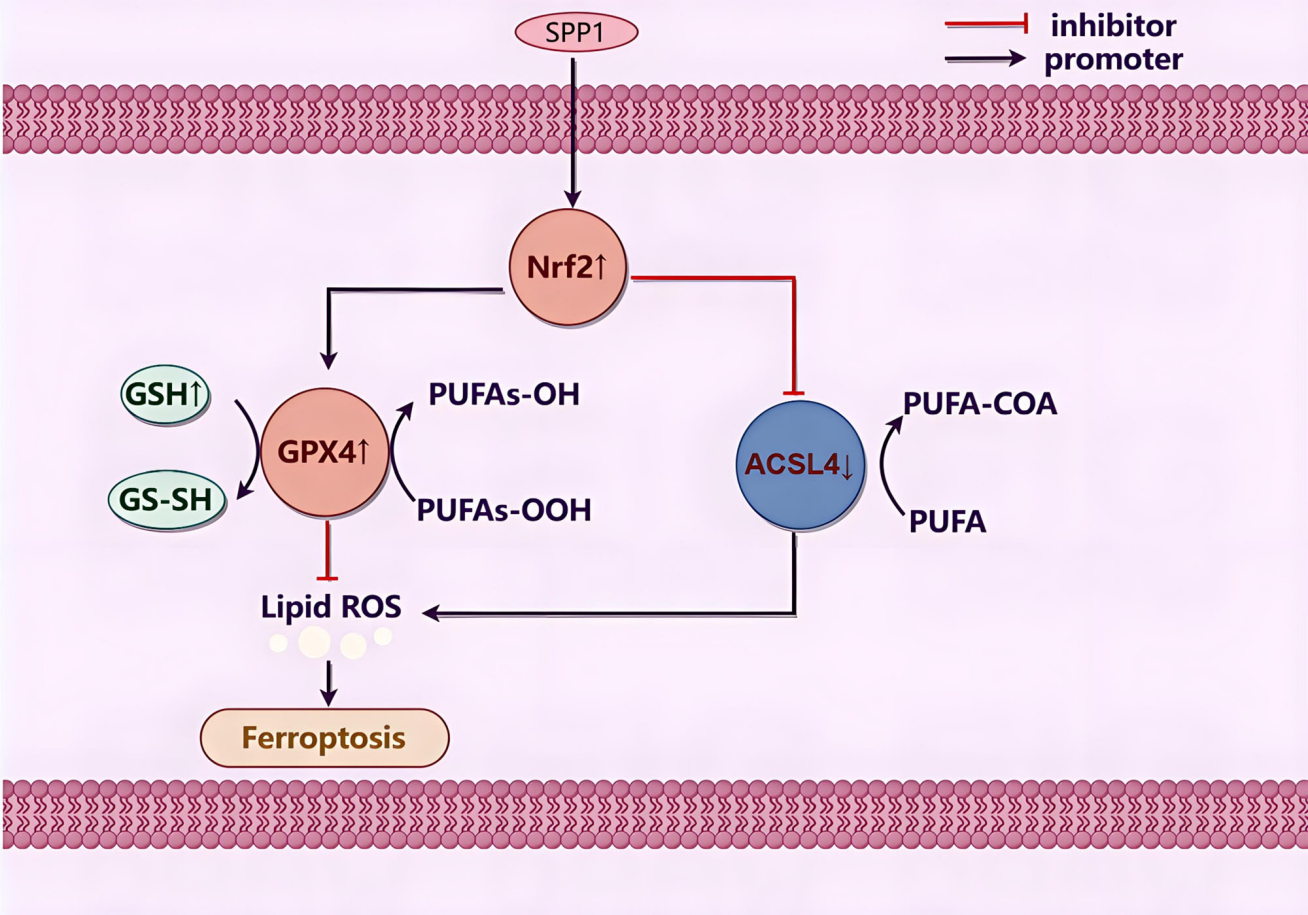
OPN reduces ferroptosis by promoting GPX4 and inhibiting ACSL4 through the Nrf2 pathway.

Given the nascent research on the implications of ferroptosis in ICH, this study aimed to investigate the role of OPN in ICH‐associated ferroptosis. Exploring OPN function in this context is crucial because it interacts with multiple receptors, including integrins and CD44 variants, and acts as a complex adhesion protein and cytokine (Tong et al. 2024). This study aimed to uncover the potential ability of OPN to modulate ferroptosis, a form of cell death characterized by iron‐dependent lipid peroxidation that is important in the pathophysiology of ICH (Li et al. 2020). By understanding how OPN influences ferroptosis, this study aimed to contribute to the development of novel therapeutic strategies for ICH, a condition for which current treatments are limited and often inadequate(figure 1).

## 2 Materials and Methods

### 2.1 Microarray Data

The clinical data of patients with ICH were obtained from the GEO database(URL: https://www.ncbi.nlm.nih.gov/geo/), specifically from Rosell GSE24265 dataset (Rosell et al. 2011). Within the GSE24265 dataset, microarray data were obtained from brain samples from four individuals with ICH. The collection encompassed 11 samples comprising both perihematomal tissue and matching areas on the opposite side of the brain, including white and gray matter. For the purposes of this anonymized data retrieval and analysis study, neither patient consent nor ethical committee approval was required.

### 2.2 The FerrDb Database

The FerrDb database (http://www.zhounan.org/ferrdb/), a comprehensive repository of ferroptosis‐related genes, was utilized to identify ferroptosis drivers, suppressors, and markers. This database collates experimentally validated genes and pathways associated with ferroptosis from published studies, providing an updated resource for mechanistic and biomarker research in this field.

### 2.3 Differential Expression Analysis

The study employed the limma package (version 2.10; URL: https://bioconductor.org/packages/2.10/bioc/html/limma.html) in R (version 3.18.0; URL: https://cran.r‐project.org/bin/windows/base/old/). The limma software was used to scrutinize the GEO dataset for differentially expressed mRNAs. The threshold for considering mRNAs as differentially expressed was set at a significance level of adjusted *p*‐value < 0.05 and a logarithmic fold‐change threshold of greater than 1 or less than –1. A functional enrichment analysis was performed to decipher the underlying roles of these potential targets. This analysis leveraged a Gene Ontology (GO) enrichment analysis to categorize genes based on their molecular function, involvement in biological pathways, and location within cellular components. Additionally, a Kyoto Encyclopedia of Genes and Genomes (KEGG; URL: https://www.genome.jp/kegg/) enrichment analysis was used to delve into the gene functions and extract insights about key functions (Kanehisa et al. 2024; Kanehisa 2019; Kanehisa 2000).

To ensure a thorough understanding of the role of mRNAs in the disease pathology of ICH, the GO (URL: https://www.geneontology.org/) functions of the potential targets were meticulously examined and KEGG pathways enriched using the Cluster Profiler software package in R. The pheatmap package in R was used to generate a heat map for visual representation. Data from the FerrDb(version FerrDb V2;URL: http://www.zhounan.org/ferrdb/)repository at zhounan.org were integrated with the GSE24265 dataset to pinpoint DEGs linked to ferroptosis. The Venny 2.1(URL: https://bioinfogp.cnb.csic.es/tools/venny/index.htm) online tool was then used to create a Venn diagram that highlighted the intersecting areas between the datasets. DEGs associated with ferroptosis were subsequently uploaded to Metascape (version v3.5.20240901; URL: http://metascape.org/gp/index.html) an online platform for gene function annotation analysis, for annotation of the biological processes. This analysis focused on genes that were common to the GSE24265 dataset and those related to iron‐induced apoptosis. An enrichment analysis of the biological pathways involving miRNAs was conducted using FunRich (version 3.1.4; URL: http://www.funrich.org/i) differences were considered statistically significant at a threshold of *p* < 0.05 (Zhou et al. 2019).

### 2.4 Protein–Protein Interaction Network Analysis

Researchers have employed Metascape, an online analytical tool designed for gene function annotation, to delineate biological processes and scrutinize genes exhibiting differential expression linked to iron‐mediated cell death. This approach facilitates a comprehensive analysis of the genetic factors involved in ferroptosis and provides insights into the molecular mechanisms underlying this form of cell demise (Szklarczyk et al. 2017). For our analysis, we focused on the intersection of genes present in both the GSE24265 dataset and the datasets related to ferroptosis. We utilized the STRING database (version 11.0, https://string‐db.org/) and Cytoscape (version 3.10.2, https://cytoscape.org/) to construct a protein interaction network. Next, we employed the Molecular Complex Assay (MCODE) to cluster the gene networks, which allowed us to identify crucial modules within the protein–protein interaction (PPI) network (Bader and Hogue 2003). MCODE (version 2.0.0) is primarily used to detect and extract crucial subnetworks or modules within a larger PPI network. These modules, each associated with distinct biological functions, were defined by varying the module scores of the genes they contained. These scores were used to identify key genes within the network. A threshold of *p* < 0.05 was applied to determine which modules were significantly different, highlighting the most impactful differences.

### 2.5 Animals

Male C57BL/6J mice aged 8–12 weeks were maintained in a regulated environment at 23 ± 1°C, humidity at 40%–60%, and a 12‐h light‐dark cycle. The animals were group‐caged (three to five each) with unlimited access to food and water. All mice used herein were obtained from the Animal Laboratory of Jiangnan University. All animal protocols were approved by the Animal Ethics Committee of The Affiliated Wuxi No. 2 People's Hospital (2023 Y202). This study is performed in accordance with relevant guidelines and regulations. All methods are reported in accordance with ARRIVE guidelines. Randomization of subjects was achieved via computer‐generated allocation, and investigators performing outcome assessments were blinded to group assignments. Sample sizes were determined a priori using power analysis (α = 0.05, power = 0.8), and welfare protocols followed institutional standards.

### 2.6 ICH Model

The first stage involved establishing a mouse model of ICH by injecting collagenase IV into the striatum. A precise volume of 2.5 µL of a 0.9% saline solution, enriched with 0.03 U of collagenase IV, was administered at a rate of 0.18 µL/min into the left striatum at a depth of 3.2 mm under stereotaxic guidance. After the infusion, the needle was retained for an additional 10 min to prevent reflux. Subsequently, the microsyringe was carefully withdrawn and the skull opening sealed with bone wax and sutures. The experimental mice were then placed on an electric heating pad to maintain their body temperature at 37°C for postoperative recovery (Xu et al. 2022). The mice were then randomly assigned to one of three groups: sham, ICH, or ICH + OPN. The sham group was subjected to identical surgical procedures as the experimental groups but without the collagenase administration.

### 2.7 Nasal Administration of OPN

OPN 5 µg was administered intranasally as nose drops (5 µL/drop) over 20 min. The drops were alternated every 2–5 min between the left and right nares. For neuroprotection studies, a total volume of 50 µL was administered. Treatment with OPN was initiated 10 min after the onset of ICH. This protocol was adapted from previous studies that demonstrated the therapeutic potential of OPN in stroke models (Doyle et al. 2008).

### 2.8 Neurological Deficit Assessments (Longa Score)

The Longa scoring system may be summarized as follows: 0, no neurological deficits; 1, partial extension failure of the forelimbs and minor neurological deficits; 2, side flipping and moderate neurological deficits while walking; 3, side jumping and severe neurological deficits; and 4, no response to noxious stimuli. All score evaluations were conducted by two impartial researchers who were unaware of the group assignments.

### 2.9 Magnetic Resonance Imaging

Hematoma size was assessed using a magnetic resonance imaging (MRI) scanner (Bruker BioClinScan) (256 × 256 matrix, 1‐mm slice thickness, TR = 3000 ms, TE = 40 ms). Hematomas were identified as hyperintense regions on the MRI, manually outlined using ImageJ (version 1.54m; URL: https://imagej.net/ij/download.html) software, and quantified by summing the areas across slices and multiplying by the slice thickness. Hematoma regions were manually delineated on each slice using ImageJ software. To ensure accuracy, this process was independently performed by two experienced researchers, and inter‐rater reliability was assessed using the intraclass correlation coefficient (ICC > 0.95). Semi‐automated thresholding was applied based on the signal intensity difference between the hematoma and surrounding tissue. The threshold range was set at 1.5–2.5 times the average signal intensity of normal brain tissue to effectively distinguish the hematoma from adjacent structures.

### 2.10 Perls Prussian Blue Staining

Prussian blue staining was used to detect ferroptosis in perihematomal brain tissue post‐ICH that manifested as a distinctive blue coloration. Paraffin‐embedded sections were first cleared of wax using toluene and then rehydrated through a graded ethanol series. The samples were then treated with Perls Prussian blue stain for 15 min, followed by rinsing with distilled water to remove any unbound stain and brief counterstaining with hematoxylin for 30 s. After a series of ethanol dehydration steps and clearance with xylene, the sections were mounted in a neutral resin. Finally, the stained samples were examined under a microscope (DMI8; Leica,, Germany).

### 2.11 Western Blotting

Proteins were extracted from the perihematomal tissue using RIPA buffer supplemented with a protease inhibitor (Beyotime, China). Protein concentrations were measured using a BCA kit (CwBio, China). Each sample containing 40 µg of protein was separated by electrophoresis on a 12% sodium dodecyl sulfate–polyacrylamide gel electrophoresis gel and then transferred to polyvinylidene fluoride membranes. The membranes were blocked with Tris‐buffered saline with 20% Tween containing 5% nonfat milk for 1 h and incubated with primary antibodies at 4°C overnight including GPX4 (1:1000; cat. #ab175186; Abcam, USA), ACSL4 (1:1000; cat. #A14192; Abclonal,, China), and glyceraldehyde 3‐phosphate dehydrogenase (GADPH; 1:10,000; cat. #60004‐1‐Ig; Proteintech,, China). After extensive washing, the membranes were incubated with horseradish peroxidase–conjugated secondary antibodies (1:5000; Proteintech, China; cat # SA00001‐1 and # SA00001‐2) for 1 h at room temperature. Protein detection was performed using the Bio‐Rad ChemiDoc XRS+ system, and band optical density was quantified using ImageJ software. Data were normalized to the optical density of GADPH and are expressed as the ratio of target protein to GADPH.

### 2.12 Biochemical Testing of Ferroptosis‐Related Indicators

Once the ICH model was successfully established, the brain tissue was rinsed with normal saline to eliminate any blood from its surface. The tissue was then weighed, homogenized in phosphate‐buffered saline at 4°C, and centrifuged at 10,000 × *g* for 10 min at 4°C. The supernatants were stored on ice for subsequent analyses. The concentrations of malondialdehyde (MDA), glutathione (GSH), and superoxide dismutase (SOD) were determined using Solarbio kits according to the manufacturer's protocols. The optical density of each parameter was measured using a microplate reader after addition of the respective reagents. The protein content of the remaining samples was determined using a BCA kit (CwBio). The levels of these parameters were calculated using the formula: (Δ*A*–*b*) / *a* × ( *f*/*Cpr*).

### 2.13 Electron Microscopy Methodology

Samples were prepared by fixation and embedding, followed by imaging using scanning electron microscopy (SEM) or transmission electron microscopy (TEM). For three‐dimensional reconstruction, multiple projections were captured and processed using Fourier transform techniques to visualize the structures at high resolution.

### 2.14 Statistical Analysis

Numerical analyses were performed using GraphPad Prism and SPSS 22.0. Student's unpaired two‐tailed *t*‐tests were used to evaluate statistical intergroup differences. In scenarios involving multiple comparisons without rank measures, a one‐way analysis of variance was conducted, followed by a post hoc analysis using Tukey's method. Survival rates were statistically assessed using the log‐rank (Mantel–Cox) test. Data are expressed as mean ± SEM, and values of *p* < 0.05 were considered significant in all analyses.

## 3 Results

### 3.1 Differential Gene Expression Results

**FIGURE 2:**
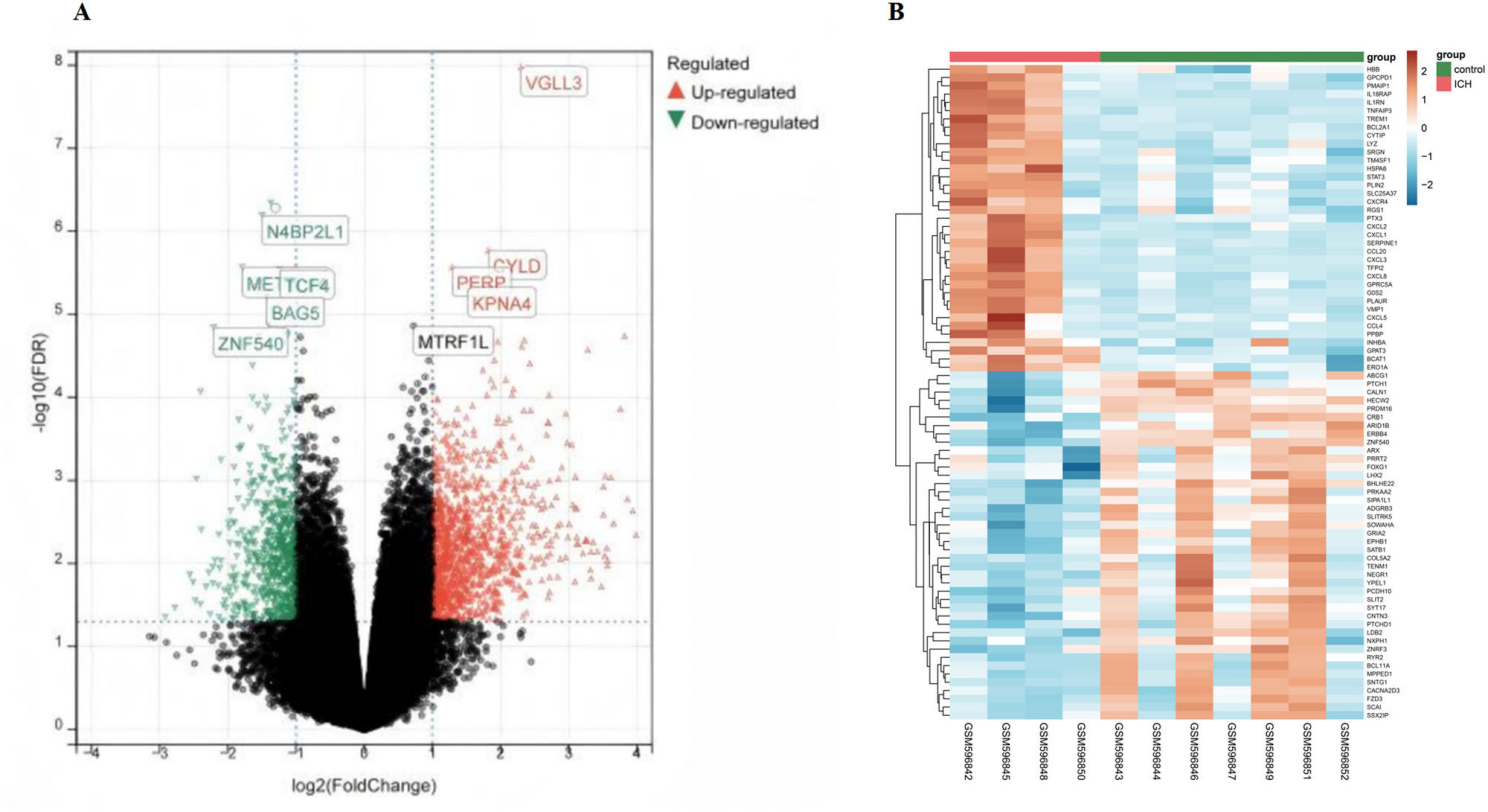
(A) Volcano plot incorporating both fold‐change and adjusted *p*‐value metrics in which the red dots indicate upregulated genes and the blue dots denote downregulated genes. (B) A heatmap of differential gene expression illustrates the expression trends across various tissues using a spectrum of colors. This heatmap features the top 50 upregulated and top 50 downregulated genes.

The GSE24265 microarray dataset retrieved from the GEO repository encompassed both white and gray matter tissues surrounding the hematoma in the four patients as well as the corresponding healthy tissues from the opposite side. A volcano plot illustrating the differential genes is shown in Figure 1, 2, with a selection of 50 upregulated and 50 downregulated genes used to create a heat map of DEG expression (Figure 2B).

**FIGURE 3:**
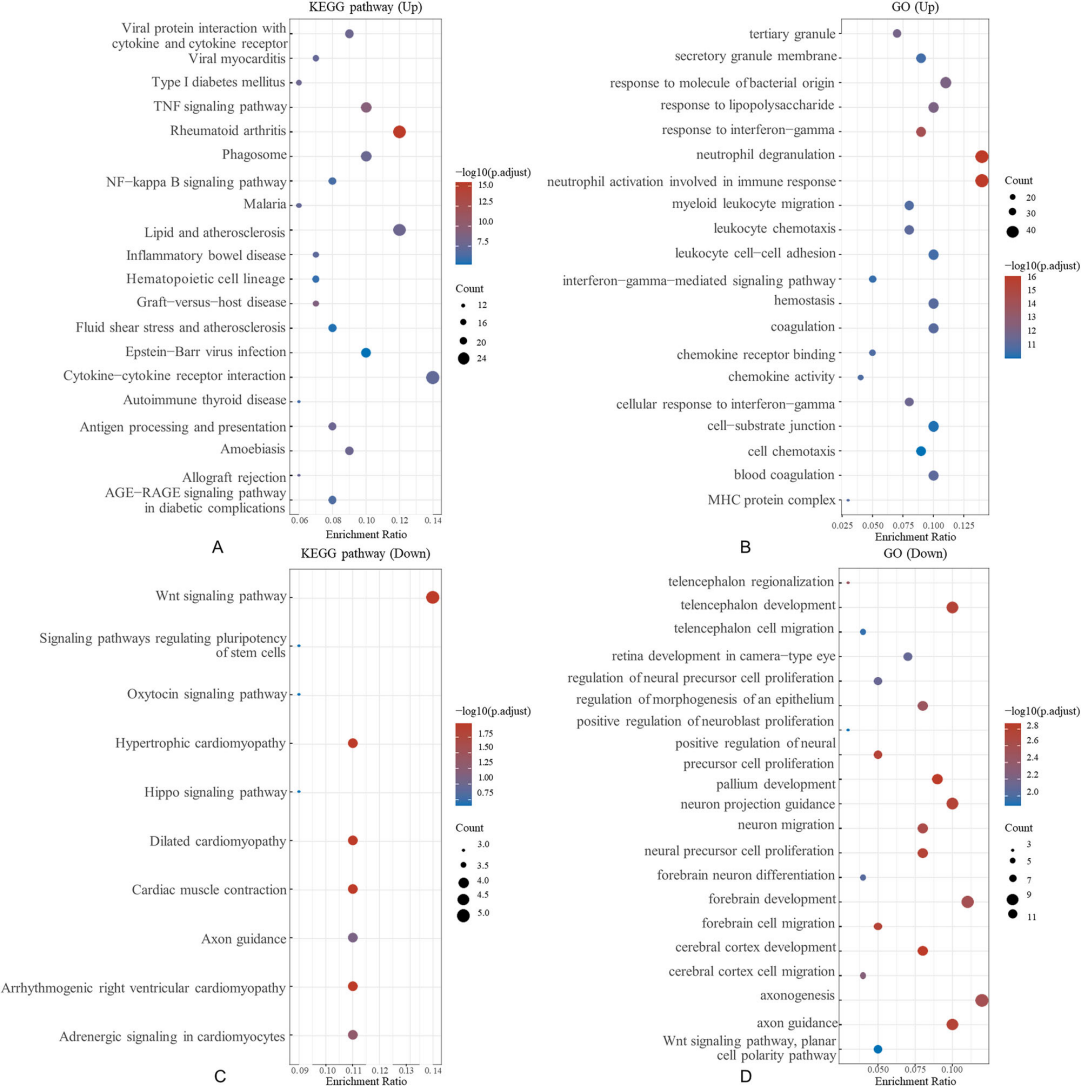
The functional enrichment analysis employed enriched KEGG pathways to emphasize the key biological roles of the underlying mRNAs. The *x*‐axis shows the gene proportion, while the *y*‐axis lists the enriched pathways. For the Gene Ontology analysis of potential mRNA targets, we used the Cluster Profiler package in R software (version 3.18.0) to classify biological processes, cellular components, and molecular functions. Color intensity in the visualization reflects the significance of the differential enrichment, while circle size denotes the number of genes, with larger circles indicating a higher gene count. Pathways with a false‐discovery rate < 0.05 were deemed significant in the enrichment analysis (enrichment score of –log10 (*p*) > 1.3).

We subsequently performed KEGG and GO analyses of the DEGs. We subsequently performed KEGG and GO analyses of the DEGs. The main upregulated category in KEGG was *viral protein interaction with cytokine and cytokine receptor* (Figure 3A), while the main downregulated categories were *the Wnt signaling pathway* (Figure 3B). The main upregulated categories in GO were t*ertiary granule* (Figure 3C). The main downregulated categories in GO were *telencephalon regionalization* (Figure 3D).

### 3.2 Ferroptosis‐Associated DEGs

**FIGURE 4:**
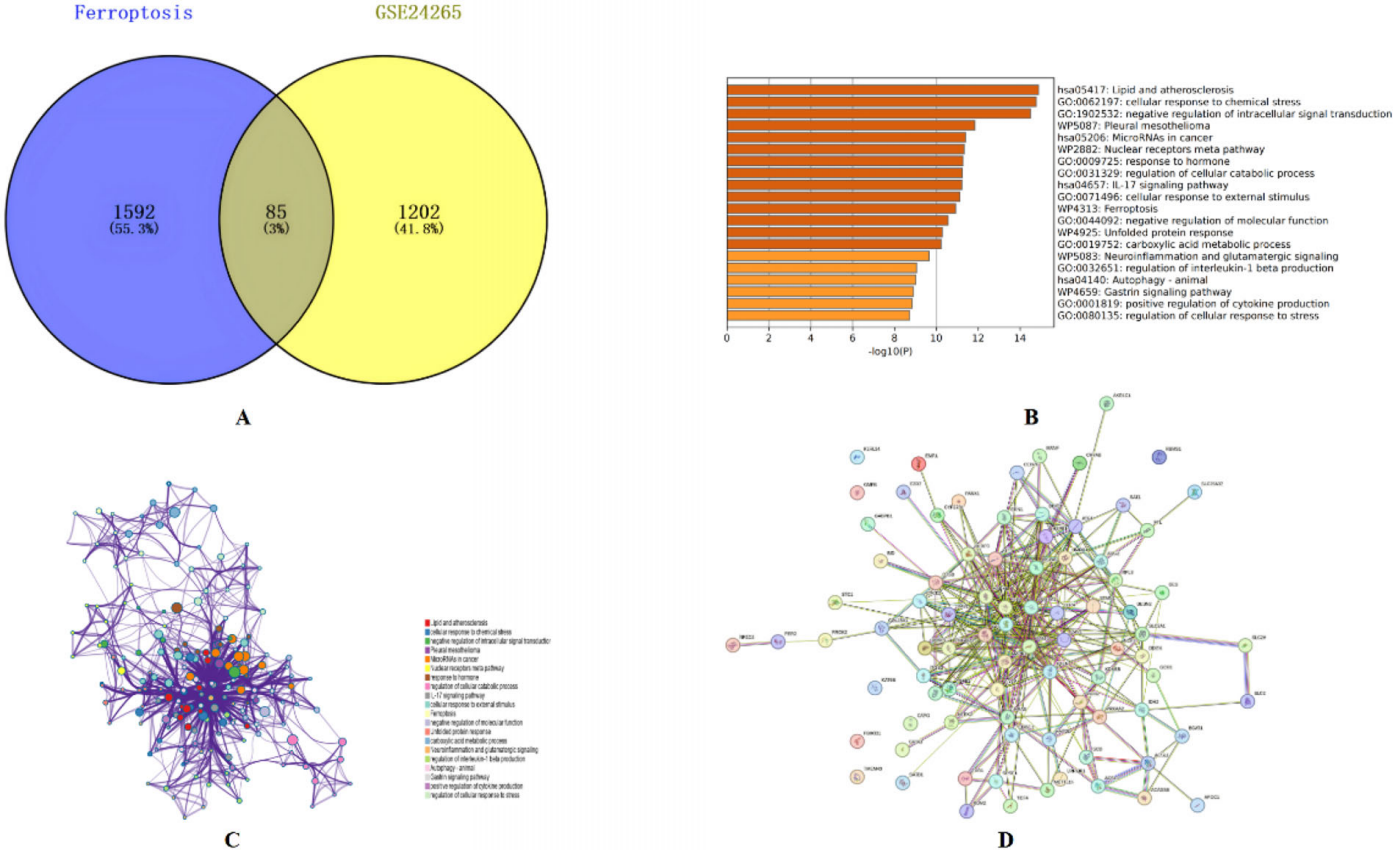
(A) The Venn diagram illustrates the intersection of differentially expressed genes. We integrated the ferroptosis data from FerrDb with the GSE24265 dataset to assess the degree of ferroptosis. (B) A Metascape analysis produced a bar graph depicting 20 biological pathways prioritized by *p*‐values and gene percentages (statistical significance, *p* < 0.01). The findings point to substantial enrichment in biological processes associated with the response to oxidative stress. Figure 3B is based on the 85 DEGs identified in Figure 3A. (C) In Metascape, an interactome network was constructed for 85 differentially expressed iron‐related genes. (D) This step fortified the network of the associated provisions.

Researchers identified a dataset comprising 85 DEGs associated with ferroptosis in ICH by intersecting DEGs with genes related to iron death in the FerrDb database (Figure 4A). Of these, 62 were upregulated and 23 were downregulated. These DEGs related to ICH ferroptosis were then subjected to a Metascape analysis, which revealed significant activation of biological pathways including Lipid and Atherosclerosis, Cellular Response to Chemical Stress, Negative Regulation of Intracellular Signal Transduction, and Pleural Mesothelioma (Figure 4B, C). Subsequently, the DEGs associated with ferroptosis in ICH were imported into the STRING database for the construction of a PPI network for the ferroptosis‐related DEGs using the default cutoff for network construction (interaction score > 0.4) (Figure 4D).

### 3.3 PPI Network Analysis of Ferroptosis‐Related DEGs in ICH

**FIGURE 5:**
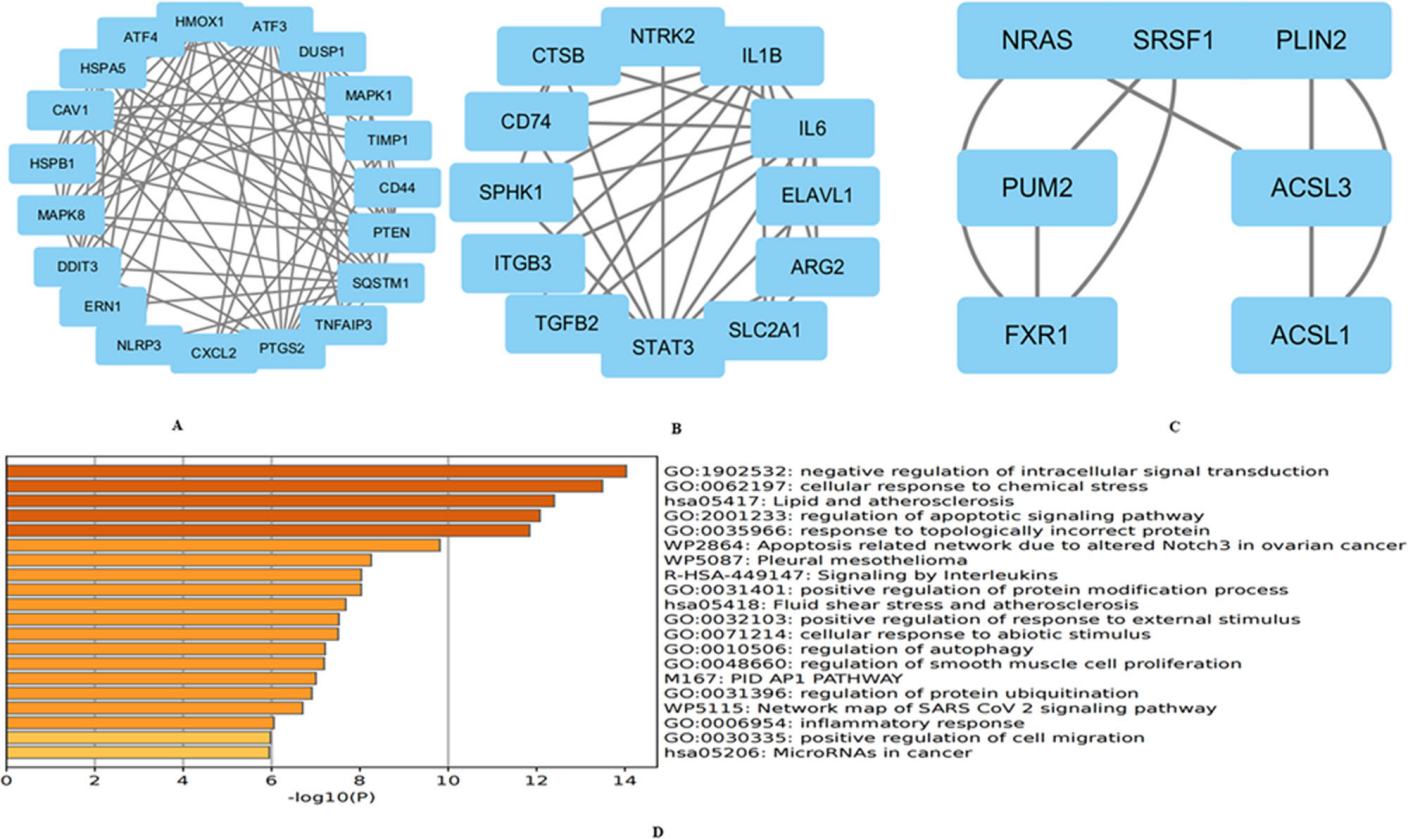
The Cytoscape network visualized 85 nodes, displaying an interaction score of > 0.4 sourced from the STRING online digital library. Each node represents a gene, and the edges represent the connections between them. The Molecular Complex Assay algorithm identified three key modules for network gene clustering. (A) Cluster 1. (B) Cluster 2. (C) Cluster 3. (D) Functional enrichment analysis of cluster 1.

Researchers imported the PPI network from the STRING database into Cytoscape software and applied the MCODE algorithm to identify three significant modules consisting of 27 key genes (Figures 5A–C). Among them, two SPP1 receptors, CD44 and ITGB3, were identified, sparking interest in the potential role of OPN in ferroptosis‐associated DEGs in ICH, a relationship not previously documented.

Furthermore, an analysis of the top cluster from Metascape revealed that these genes were predominantly involved in the negative regulation of intracellular signal transduction, cellular responses to chemical stress, and pathways related to lipid metabolism and atherosclerosis (Figure 5D).

### 3.4 OPN Significantly Improves Neurological Deficits Post‐ICH

**FIGURE 6:**
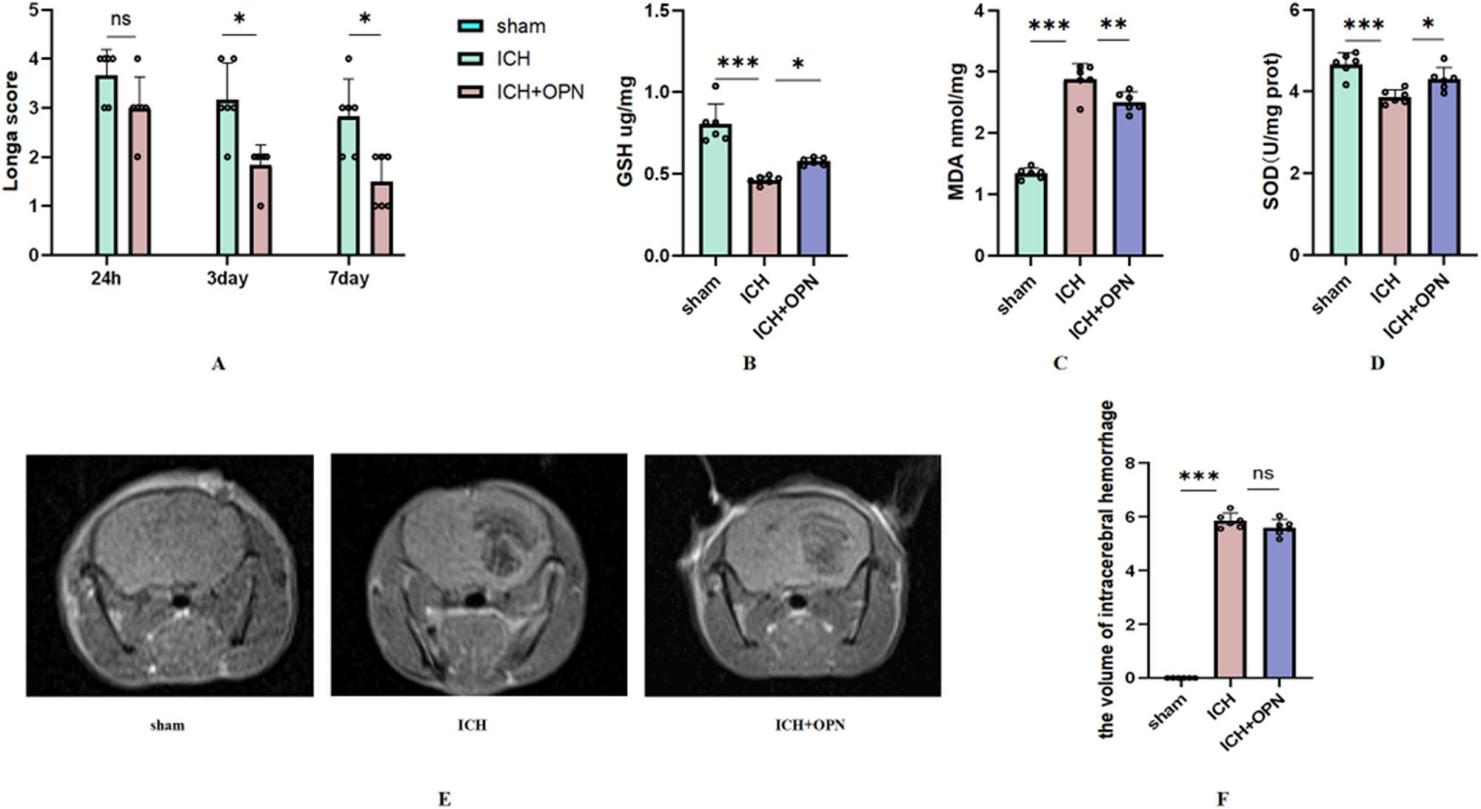
(A) Neurological recovery was evaluated using Longa scores on days 1, 3, and 7 following intracerebral hemorrhage (ICH) (*n* = 6 mice/group; repeated measures analysis of variance [ANOVA] followed by Bonferroni's post hoc test with results expressed as mean ± standard deviation). Statistical significance is denoted as follows: *p < 0.05, **p < 0.01, ***p < 0.001, and ****p < 0.0001. (B) Glutathione (GSH) levels in the perihematomal tissue were measured for all treatment groups on day 3, and differences were analyzed using one‐way ANOVA with Tukey's post hoc test (*n* = 6 mice/group), *F* = 34.25. Significance levels are indicated as *p < 0.05, **p < 0.01, ***p < 0.001 versus sham group and *p < 0.05, **p < 0.01, and ***p < 0.001 versus ICH group. (C) Malondialdehyde (MDA) levels in the perihematomal tissue were similarly assessed across treatment groups on day 3 using one‐way ANOVA and Tukey's post hoc test (*n* = 6 mice/group), with significance represented as in (B). *F* = 115.12. (D) Superoxide dismutase (SOD) levels in the perihematomal tissue were analyzed on day 3 (*n* = 6 mice/group), with significance noted as in (B). *F* = 15.22 (E) Axial MRI image of a murine brain taken at 24 h after collagenase‐induced ICH. A hyperintense area is visible in the left striatum representing the hemorrhagic lesion. (F) Quantitative analysis of hematoma size in the ICH versus ICH+OPN groups. Data are presented as mean ± SEM (*n* = 6 mice/group), *F* = 135.31 Although the mean hematoma size in the ICH+OPN group appears smaller than that in the ICH group, the difference is not statistically significant (*p* > 0.05, one‐way ANOVA and Tukey's post hoc test), suggesting that the reduction in hematoma size in the ICH+OPN group may not have biological significance in 24 h.

Once an ICH model was established, the effects of OPN on neurological outcomes were evaluated using Longa scores in an ICH model. The ICH and ICH+OPN groups exhibited neurological deficits, but on days 3 and 7 post‐ICH, the Longa scores were markedly lower in the ICH+OPN versus ICH group, indicating that OPN may reduce ICH‐associated brain damage (Figure 6A).

Oxidative stress, a key contributor to post‐ICH brain injury, was assessed by measurement of GSH and SOD levels, which were notably lower in the ICH versus sham group (Figure 6B, D). Treatment with OPN counteracted these effects. Additionally, MDA, a marker of lipid peroxidation (Wang and Liu 2025), was elevated in the ICH group but reduced by SPP1 treatment (Figure 6C). Using MRI, we measured the hematoma area in the mouse model of ICH (Figure 6E). At 24 h post‐ICH, no statistically significant differences were observed in hematoma size between the ICH and ICH+OPN groups (Figure 6F). In conclusion, our findings suggest that OPN ameliorates ICH‐induced brain injury, potentially by inhibiting oxidative stress.

### 3.5 OPN Plays Significant Protective Role in Neuronal Ferroptosis

**FIGURE 7:**
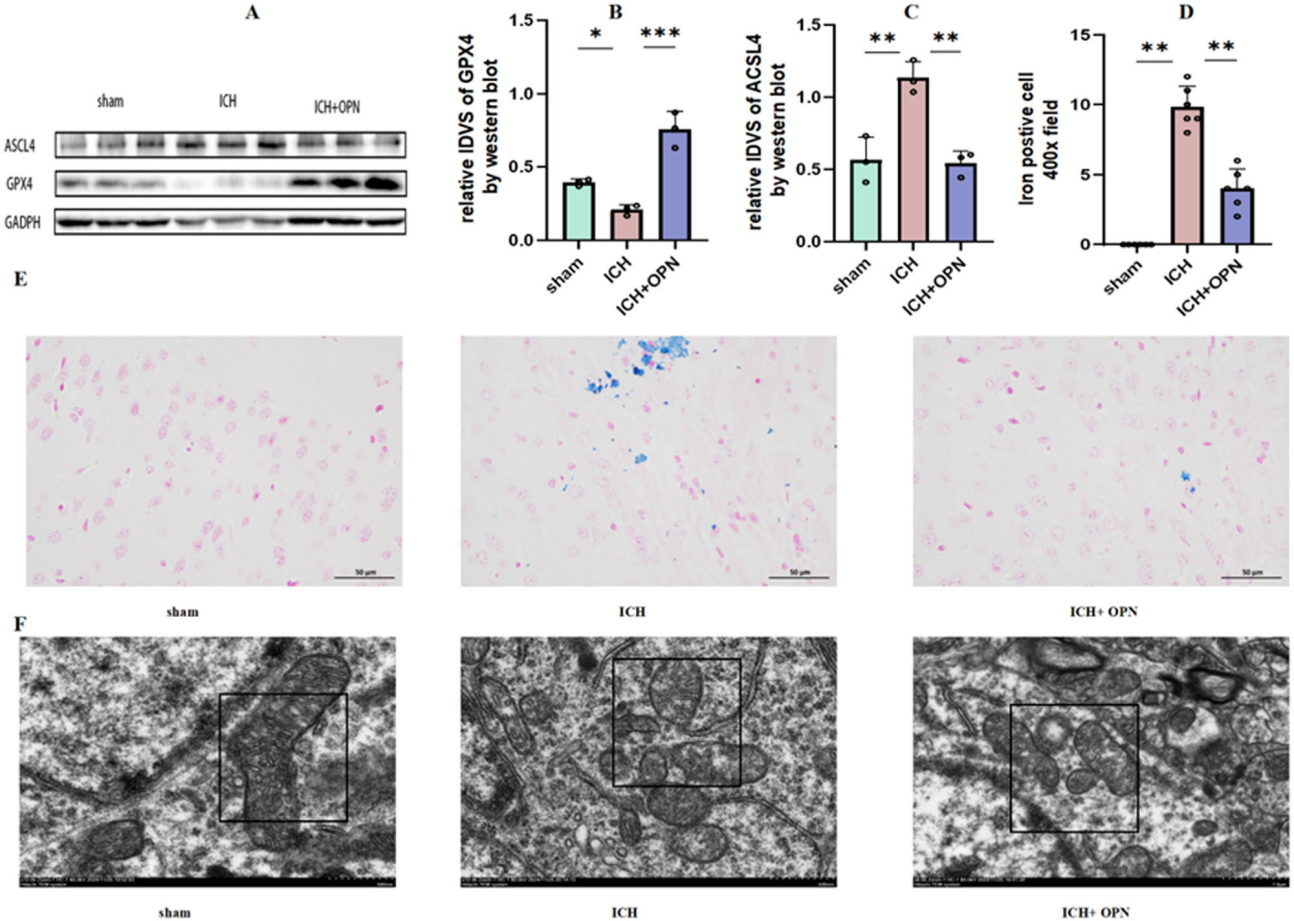
(A–D) Western blotting images showing the expression levels of *GPX4* and *ACSL4* in the perihematomal tissue on day 3 following intracerebral hemorrhage. A quantitative analysis of protein levels was performed using densitometry and normalized to β‐actin as a loading control. Data are presented as mean ± SEM (*n* = 6 mice/group). *F* = 42.18, 22.12, respectively. A statistical analysis was performed using one‐way analysis of variance followed by Tukey's post hoc test. *p < 0.05, **p < 0.01, ***p < 0.001, and ****p < 0.0001. (E) Iron distribution in the perihematomal region detected by Prussian blue staining. The scale bar represents 50 µm. Quantitative analysis of iron‐positive areas was performed using image analysis software. Data are presented as mean ± SEM (*n* = 6 mice/group). A statistical analysis was conducted using one‐way analysis of variance followed by Tukey's post hoc test. *F* = 105.62. (F) Transmission electron microscopy image of the ultrastructural features of brain tissue sections with focus on mitochondrial morphology. Mitochondria were identified and highlighted in the images, providing detailed insights into their structural integrity and distribution within the tissues (magnification, 5000×).

On day 3 post‐ICH, western blot analysis demonstrated a significant downregulation of the ferroptosis‐related gene GPX4 and a concurrent upregulation of ACSL4. Treatment with OPN effectively modulated the expression of these ferroptosis‐related genes, suppressing ACSL4 while enhancing GPX4 expression, thereby attenuating cellular ferroptosis (all *p* < 0.05; Figure 7A–D). Moreover, Prussian blue staining revealed that OPN notably decreased ICH‐associated iron accumulation (Figure 7E).

TEM was used to examine the ultrastructure of the brain samples. Under normal conditions, the mitochondrial membranes were distinct with clearly visible cristae. However, post‐ICH, the mitochondria became swollen, the cristae disappeared, and cytoplasmic vacuolization was evident. These changes suggest that ultrastructural damage is indicative of ferroptosis in neurons. However, the presence of OPN mitigated this damage (Figure 7F).

### 3.6 OPN Exerts Ferroptosis‐Protective Effects via Nrf2/HO1 Pathway

Next, we explored the mechanisms underlying the effects of OPN on ferroptosis. Given that the PI3K/AKT pathway, commonly associated with OPN, can mitigate ferroptosis, we hypothesized that OPN binds to its receptors to activate it. This activation could, in turn, inhibit the expression of ferroptosis‐related genes, such as *ACSL4* and *GPX4*, thereby reducing cellular ferroptosis. Additionally, activation of the PI3K/AKT pathway enhances the cellular antioxidant system, further strengthening cellular resistance to ferroptosis.

**FIGURE 8:**
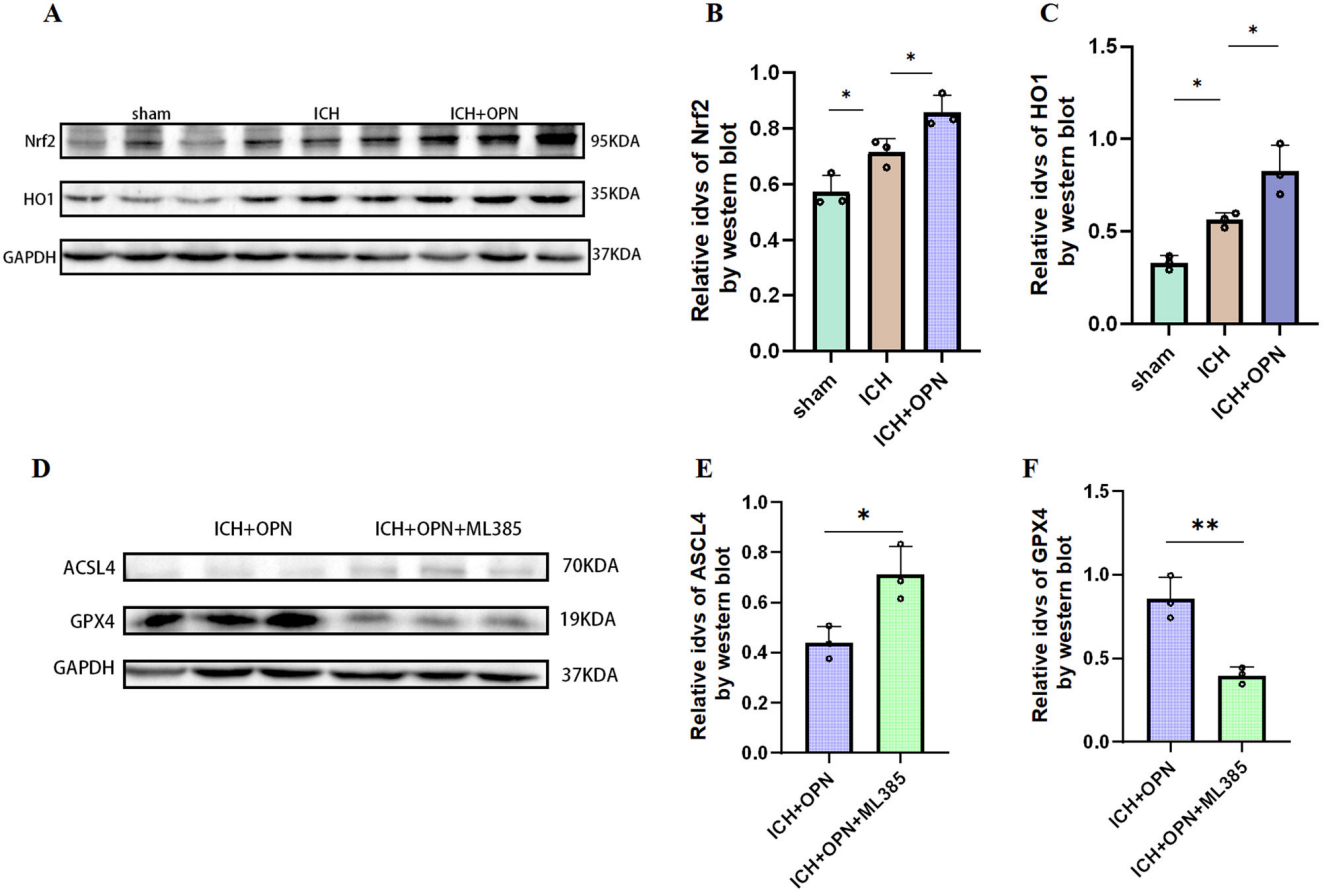
(A–C) Western blotting images showing the expression levels of Nrf2, HO1 in brain tissue lysates from the ICH and OPN+ICH groups. Compared with the ICH group, the OPN+ICH group exhibited increased levels of Nrf2 and HO1, indicating enhanced activation of the Nrf2/HO1 pathway. Data are presented as mean ± SEM (*n* = 6 mice/group). A statistical analysis was performed using one‐way analysis of variance followed by Tukey's post hoc test. *F* = 20.14, *F* = 25.43, respectively; *p < 0.05, **p < 0.01 versus ICH group. (D– F) Western blotting images showing the expression levels of GPX4, ACSL4 in brain tissue lysates from the ICH+OPN and ICH+OPN+ML385 groups. Compared with the ICH+OPN group, the ICH+OPN+ML385 group exhibited decreased levels of GPX4. Data are presented as mean ± SEM (*n* = 6 mice/group). A statistical analysis was performed using unpaired *t*‐test analysis. *p < 0.05, **p < 0.01 versus ICH+OPN group.

To verify this hypothesis, we performed western blotting. Compared with the ICH group, the OPN+ICH group showed increased levels of Nrf2/HO1 (Figures 8A–C). After using the Nrf2 inhibitor ML385, Western blotting showed decreased levels of GPX4 and ACSL4, indicating that OPN's role in alleviating ferroptosis is achieved through the Nrf2 pathway(Figure 8D–F).These findings suggest that OPN plays an important protective role in regulating ferroptosis and may serve as a new therapeutic target for related diseases.

## 4 Discussion

This study pinpointed critical genes associated with ferroptosis and explored their roles in the context of ICH. We identified 85 DEGs (23 downregulated, 62 upregulated). Using the Metascape online platform, we integrated DEGs from the GSE24265 dataset with those from the FerrDb database and performed a pathway enrichment analysis, which selected 27 key genes for further analysis. Our bioinformatics‐driven approach sheds light on the potential pathological mechanisms underlying ICH. Notably, *CD44* and *ITGB3*, receptors for OPN, play a role in suppressing ferroptosis. As a secreted glycoprotein, OPN likely reduces oxidative stress and inflammation in the perihematomal region, thereby indirectly reducing ferroptotic vulnerability. OPN activates the PI3K/AKT pathway (Liu et al. 2024), which inhibits lipid peroxidation, a hallmark of ferroptosis (Su et al. 2024). Furthermore, previous studies on triple‐negative breast cancer demonstrated that OPN activates the PI3K/AKT/mammalian target of rapamycin pathway, thereby modulating GPX4 levels and combating lipid peroxidation (Guo et al. 2024). The PI3K/Akt pathway plays a significant role in the regulation of Nrf2 activity. Upon activation, PI3K phosphorylates and activates Akt, which in turn enhances Nrf2 signaling (Mukherjee et al. 2025). By assessing the activity of the Nrf2/HO‐1 signaling axis, this study systematically evaluates the biological consequences of PI3K/AKT pathway activation in osteopontin (OPN)‐mediated signaling transduction, rather than merely confirming upstream kinase phosphorylation events. This methodological design directly validates our central hypothesis that OPN exerts cytoprotective and tissue‐reparative functions through PI3K/AKT‐mediated antioxidant defense mechanisms. Although previous studies have partially characterized the fundamental role of PI3K/AKT in OPN's signaling network, the molecular cascade connecting this pathway to the Nrf2/HO‐1 antioxidant system remains a significant knowledge gap in current research. The present work innovatively elucidates a previously unrecognized regulatory mode whereby OPN maintains redox homeostasis through modulation of the Nrf2/HO‐1 axis, thereby establishing a novel conceptual framework for understanding the molecular basis of OPN‐mediated cytoprotection. This pathway can modulate the nuclear translocation of Nrf2, thereby influencing the expression of antioxidant genes such as HO‐1, NQO1, and GPX4

No studies to date have reported on the ability of OPN to alleviate ferroptosis in ICH, prompting researchers to further explore this concept. The current focus of ICH research is on secondary brain injury, with increasing evidence indicating the involvement of oxidative stress in the pathological process of post‐ICH brain injury (Guo et al. 2024). Recent studies suggested that ferroptosis, a type of nonapoptotic programmed cell death that occurs after ICH, causes secondary cell death (Sun et al. 2023). However, its underlying precise molecular mechanisms are not well understood. Ferroptosis is marked by disruptions in iron ion homeostasis, glutathione depletion, reactive oxygen species accumulation, lipid peroxidation injury, and *GPX4* suppression (Chen et al. 2021; Deng et al. 2023). Lower GPX4 levels and a diminished glutathione ratio lead to unresolved lipid peroxidation.

It is important to note that this study has certain limitations. First, our proficiency at performing mechanistic studies may be limited; thus, a comprehensive exploration of the underlying mechanisms is warranted. Second, multifaceted investigations are warranted to fully delineate OPN's mechanisms in ferroptosis mitigation. The experimental framework should be enhanced by: (1) incorporating ferroptosis inhibitors (e.g., Ferrostatin‐1) as pharmacological positive controls; (2) implementing TUNEL staining to differentiate apoptotic cell death patterns; and (3) assessing postsynaptic density protein 95 (PSD95) levels to evaluate synaptic integrity in neuronal models. Temporally, while current data focus solely on Day 3 markers, the dynamic progression of ferroptosis necessitates multi‐point analyses spanning early‐phase (24 h), intermediate (72 h), and late‐stage (5–7 days) intervals. This temporal expansion, coupled with complementary histopathological (TUNEL) and synaptic ultrastructure (PSD95) evaluations, would comprehensively characterize both morphological and functional outcomes. Such methodological enhancements would not only clarify potential crosstalk between ferroptotic and apoptotic pathways but also establish critical structure‐function correlations in synaptic degeneration mechanisms, ultimately strengthening the mechanistic conclusions. OPN's ability to cross the blood‐brain barrier (BBB) is limited by its large molecular size and hydrophilic nature, necessitating advanced delivery strategies like nanoparticle systems, BBB‐penetrating peptides, or focused ultrasound to enhance brain targeting. Additionally, optimizing pharmacokinetic properties such as bioavailability, half‐life, and tissue distribution is crucial to ensure its therapeutic efficacy in treating neurological disorders.

In the discussion of SPP1's therapeutic potential, it is important to address its comparative efficacy relative to existing therapies for intracerebral hemorrhage (ICH), such as iron chelators. While iron chelators primarily target iron‐induced oxidative damage and secondary injury, SPP1 may offer additional benefits by modulating inflammation, promoting tissue repair, and enhancing neuroprotection. However, the comparative efficacy of SPP1 remains unclear, and head‐to‐head studies are needed to evaluate their therapeutic potential, mechanisms of action, and possible synergistic effects in improving outcomes for ICH patients. This comparison will help clarify the unique advantages and limitations of SPP1 in the context of current treatment strategies.

Here we observed that OPN considerably elevated the concentrations of GPX4, GSH, and SOD while reducing ACSL4 and MDA levels and iron deposition (Figure 7). These findings provide additional support of the notion that OPN decreases iron accumulation. Collectively, these outcomes suggest that the therapeutic benefits of OPN in ICH may be attributed to the inhibition of ferroptosis and preservation of neuronal integrity. These findings significantly increase our understanding of the molecular mechanisms underlying OPN's therapeutic effects in ICH and inform the development of innovative treatment strategies for it.

## Author Contributions

**Pengpeng Li**: software, investigation, writing–original draft. **Zhenxin Tao**: visualization, methodology, writing‐review and editing. **Yangyang Gao**: writing–original draft, writing–review and editing. **Jiajia Tian**: writing–review and editing, project administration. **YaTing Zhang**: visualization. **Wenhui Yang**: project administration. **Yilu Li**: resources. **Xudong Zhao**: funding acquisition.

## Ethics Statement

Ethical approval for research involving animals at Jiangnan University was granted in compliance with ethical standards.

## Conflicts of Interest

We declare no conflicts of interest.

### Peer Review

The peer review history for this article is available at https://publons.com/publon/10.1002/brb3.70493.

## Data Availability

This study analyzed publicly available datasets stored in FerrDb (http://www.zhounan.org/ferrdb/) and the NCBI GEO database (https://www.ncbi.nlm.nih.gov/geo/query/acc.cgi?acc=GSE24265).

## References

[brb370493-bib-0001] Bader, G. D. , and C. W. Hogue . 2003. “An Automated Method for Finding Molecular Complexes in Large Protein Interaction Networks.” BMC Bioinformatics [Electronic Resource] 4, no. 2. 10.1186/1471-2105-4-2 PMC149346 12525261

[brb370493-bib-0002] Chen, S. P. , L. Li , C. Peng , et al. 2022. “Targeting Oxidative Stress and Inflammatory Response for Blood‐Brain Barrier Protection in Intracerebral Hemorrhage.” Antioxidants & Redox Signaling 37, no. 1‐3: p. 115–134. 35383484 10.1089/ars.2021.0072

[brb370493-bib-0003] Chen, X. , J. Li , R. Kang , D. J. Klionsky , and D. Tang . 2021. “Ferroptosis: Machinery and Regulation.” Autophagy 17, no. 9: p. 2054–2081.. 32804006 10.1080/15548627.2020.1810918 PMC8496712

[brb370493-bib-0004] Deng, X. , Y. Wu , Z. Hu , et al. 2023. “The Mechanism of Ferroptosis in Early Brain Injury After Subarachnoid Hemorrhage.” Frontiers in Immunology 14: 1191826.. 37266433 10.3389/fimmu.2023.1191826 PMC10229825

[brb370493-bib-0005] Doyle, K. P. , T. Yang , N. S. Lessov , et al. 2008. “Nasal Administration of Osteopontin Peptide Mimetics Confers Neuroprotection in Stroke.” Journal of Cerebral Blood Flow and Metabolism 28, no. 6: p. 1235–1248.. 18364727 10.1038/jcbfm.2008.17 PMC6015748

[brb370493-bib-0006] Guo, M. , M. Liu , W. Li , C. Wang , L. Zhang , and H. Zhang . 2024. “Osteopontin Promotes Tumor Growth and Metastasis and GPX4‐mediated Anti‐Lipid Peroxidation in Triple‐negative Breast Cancer by Activating the PI3k/Akt/mTOR Pathway.” Journal of Cancer Research and Clinical Oncology 150, no. 3: 155.. 38526702 10.1007/s00432-024-05658-w PMC10963528

[brb370493-bib-0007] Hanley, D. F. , R. E. Thompson , M. Rosenblum , et al. 2019. “Efficacy and Safety of Minimally Invasive Surgery With Thrombolysis in Intracerebral Haemorrhage Evacuation (MISTIE III): A Randomised, Controlled, Open‐Label, Blinded Endpoint Phase 3 Trial.” Lancet 393, no. 10175: p. 1021–1032. 30739747 10.1016/S0140-6736(19)30195-3 PMC6894906

[brb370493-bib-0008] Kanehisa, M. 2000. “KEGG: Kyoto Encyclopedia of Genes and Genomes.” Nucleic Acids Research 28, no. 1: p. 27–30.. 10592173 10.1093/nar/28.1.27 PMC102409

[brb370493-bib-0009] Kanehisa, M. 2019. “Toward Understanding the Origin and Evolution of Cellular Organisms.” Protein Science 28, no. 11: p. 1947–1951. 31441146 10.1002/pro.3715 PMC6798127

[brb370493-bib-0010] Kanehisa, M. , M. Furumichi , Y. Sato , Y. Matsuura , and M. Ishiguro‐Watanabe . 2024. “KEGG: Biological Systems Database as a Model of the Real World.” Nucleic Acids Research 53, no. D1: p. D672–D677.. 10.1093/nar/gkae909 PMC11701520 39417505

[brb370493-bib-0011] Karuppagounder, S. S. , I. Alim , S. J. Khim , et al. 2016. “Therapeutic Targeting of Oxygen‐Sensing Prolyl Hydroxylases Abrogates ATF4‐Dependent Neuronal Death and Improves Outcomes After Brain Hemorrhage in Several Rodent Models.” Science Translational Medicine 8, no. 328: 328ra29.. 10.1126/scitranslmed.aac6008 PMC5341138 26936506

[brb370493-bib-0012] Li, J. , F. Cao , H. L. Yin , et al. 2020. “Ferroptosis: Past, Present and Future.” Cell Death & Disease 11, no. 2: 88. 32015325 10.1038/s41419-020-2298-2 PMC6997353

[brb370493-bib-0013] Liu, C. X. , H. Ge , C. Shen , et al. 2024. “NOTCH3 promotes Malignant Progression of Bladder Cancer by Directly Regulating SPP1 and Activating PI3K/AKT Pathway.” Cell Death & Disease 15, no. 11: 840. 39557868 10.1038/s41419-024-07241-0 PMC11574029

[brb370493-bib-0014] Luo, T. L. , Q. Zheng, L. Shao, et al. 2022. “Intracellular Delivery of Glutathione Peroxidase Degrader Induces Ferroptosis in Vivo.” Angewandte Chemie‐International Edition 61, no. 39. e202206277. 35924720 10.1002/anie.202206277

[brb370493-bib-0015] Mukherjee, R. , R. Rana, S. Mehan , et al. 2025. “Investigating the Interplay between the Nrf2/Keap1/HO‐1/SIRT‐1 Pathway and the p75NTR/PI3K/Akt/MAPK Cascade in Neurological Disorders: Mechanistic Insights and Therapeutic Innovations.” Molecular Neurobiology Online ahead of print. . 10.1007/s12035-025-04725-8 39920438

[brb370493-bib-0016] Rosell, A. , A. Vilalta, T. García‐Berrocoso , et al. 2011. “Brain Perihematoma Genomic Profile Following Spontaneous Human Intracerebral Hemorrhage.” PLoS ONE 6, no. 2: e16750.. 21311749 10.1371/journal.pone.0016750 PMC3032742

[brb370493-bib-0017] Su, H. , C. Peng, and Y. Liu,. 2024. “Regulation of Ferroptosis by PI3K/Akt Signaling Pathway: A Promising Therapeutic Axis in Cancer.” Frontiers in Cell and Developmental Biology 12: 1372330. 38562143 10.3389/fcell.2024.1372330 PMC10982379

[brb370493-bib-0018] Sun, Y. , Q. Li , H. Guo, and Q. He,. 2023. “Ferroptosis and Iron Metabolism After Intracerebral Hemorrhage.” Cells 12, no. 1: 90.. 10.3390/cells12010090 PMC9818318 36611883

[brb370493-bib-0019] Szklarczyk, D. , J. H. Morris , H. Cook , et al. 2017. “The STRING Database in 2017: Quality‐Controlled Protein‐protein Association Networks, Made Broadly Accessible.” Nucleic Acids Research 45, no. D1: p. D362–D368.. 27924014 10.1093/nar/gkw937 PMC5210637

[brb370493-bib-0020] Tong, W. , T. Wang , Y. Bai , et al. 2024. “Spatial Transcriptomics Reveals Tumor‐Derived SPP1 Induces Fibroblast Chemotaxis and Activation in the Hepatocellular Carcinoma Microenvironment.” Journal of Translational Medicine 22, no. 1.. 10.1186/s12967-024-05613-w PMC11391636 39267037

[brb370493-bib-0021] Wang, Y. N. , and S. Y. Liu . 2025. “The Role of ALDHs in Lipid Peroxidation‐Related Diseases.” International Journal of Biological Macromolecules 288: 138760. 39674477 10.1016/j.ijbiomac.2024.138760

[brb370493-bib-0022] Xu, J.i , S. M. Firouz, M. Farrokhian , et al. 2022. “Potential Anti‐Inflammatory Effect of Anti‐HMGB1 in Animal Models of ICH by Downregulating the TLR4 Signaling Pathway and Regulating the Inflammatory Cytokines Along With Increasing HO1 and NRF2.” European Journal of Pharmacology 915: 174694.. 34896108 10.1016/j.ejphar.2021.174694

[brb370493-bib-0023] Zhao, X. , S.‐M. Ting, C.‐H. Liu , et al. 2017. “Neutrophil Polarization by IL‐27 as a Therapeutic Target for Intracerebral Hemorrhage.” Nature Communications 8, no. 1: 602.. 10.1038/s41467-017-00770-7 PMC5605643 28928459

[brb370493-bib-0024] Zhou, Y. , Y. Yao , L. Shen , J. Zhang , J. H. Zhang , and A. Shao . 2020. “Osteopontin as a Candidate of Therapeutic Application for the Acute Brain Injury.” Journal of Cellular and Molecular Medicine 24, no. 16: p. 8918–8929.. 32657030 10.1111/jcmm.15641 PMC7417697

[brb370493-bib-0025] Zhou, Y. Y. , B. Zhou , L. Pache , et al. 2019. “Metascape Provides a Biologist‐Oriented Resource for the Analysis of Systems‐Level Datasets.” Nature Communications 10, no. 1: 1523. 10.1038/s41467-019-09234-6 PMC6447622 30944313

